# Psychosocial factors related to sleep in adolescents and their willingness to participate in the development of a healthy sleep intervention: a focus group study

**DOI:** 10.1186/s12889-022-14278-3

**Published:** 2022-10-07

**Authors:** Ann Vandendriessche, Maïté Verloigne, Laura Boets, Jolien Joriskes, Ann DeSmet, Karlien Dhondt, Benedicte Deforche

**Affiliations:** 1 Department of Public Health and Primary Care, Faculty of Medicine and Health Sciences, Ghent University, Ghent, Belgium; 2 Clinical and Health Psychology, Université Libre de Bruxelles, Brussels, Belgium; 3 Department of Communication Studies, University of Antwerp, Antwerp, Belgium; 4 Department of Psychiatry: Pediatric Sleep Center, Ghent University Hospital, Ghent, Belgium; 5 Physical Activity, Nutrition and Health Research Unit, Faculty of Physical Education and Physical Therapy, Vrije Universiteit Brussel, Brussels, Belgium

**Keywords:** Sleep, Adolescents, Factors, Participatory research, Sleep intervention, Behavior change

## Abstract

**Background:**

Over the last decades, adolescents’ sleep has deteriorated, suggesting the need for effective healthy sleep interventions. To develop such interventions, it is important to first gather insight into the possible factors related to sleep. Moreover, previous research has indicated that chances of intervention effectivity could be increased by actively involving adolescents when developing such interventions. This study examined psychosocial factors related to sleep in adolescents and investigated adolescents’ willingness to participate in the development of a healthy sleep intervention.

**Methods:**

Nine focus group interviews were conducted with seventy-two adolescents (63.9% girls, 14.8 (± 1.0) years) using a standardized interview guide. Interviews were audio-recorded and thematic content analysis was performed using Nvivo 11.

**Results:**

Adolescents showed limited knowledge concerning sleep guidelines, sleep hygiene and the long-term consequences of sleep deficiency, but they demonstrated adequate knowledge of the short-term consequences. Positive attitudes towards sleep were outweighed by positive attitudes towards other behaviors such as screen time. In addition, adolescents reported leisure activities, the use of smartphones and television, high amounts of schoolwork, early school start time and excessive worrying as barriers for healthy sleep. Perceived behavioral control towards changing sleep was reported to be low and norms about sufficient sleep among peers were perceived as negative. Although some adolescents indicated that parental rules provoke feelings of frustration, others indicated these have a positive influence on their sleep. Finally, adolescents emphasized that it would be important to allow students to participate in the development process of healthy sleep interventions at school, although adult supervision would be necessary.

**Conclusion:**

Future interventions promoting healthy sleep in adolescents could focus on enhancing knowledge of sleep guidelines, sleep hygiene and the consequences of sleep deficiency, and on enhancing perceived behavioral control towards changing sleep. Interventions could also focus on prioritizing positive sleep attitudes over positive attitudes towards screen time, finding solutions for barriers towards healthy sleep and creating a positive perceived norm regarding healthy sleep. Involving adolescents in intervention development could lead to intervention components that match their specific needs and are more attractive for them.

**Supplementary Information:**

The online version contains supplementary material available at 10.1186/s12889-022-14278-3.

## Background

Adolescents’ sleep has deteriorated over the last decades [1]. Although the optimal amount of sleep in adolescence is eight to ten hours per night [2], a meta-analysis of 41 international surveys estimated that 53% of adolescents reported a sleep duration of less than eight hours [3]. In addition, 20–40% of adolescents worldwide experienced daytime sleepiness and 20–26% of adolescents reported a sleep onset latency greater than 30min; these are both indicators of reduced sleep quality and quantity [3]. Recent data of the Flemish 2017/2018 Health Behavior in School-aged Children survey shows an even higher prevalence of sleep deprivation and reduced sleep quality in Flemish adolescents: 59.4% of boys and 56.0% of girls between 13- and 18-years-old report that they sleep, on average, less than eight hours on school days, and 45.5% of boys and 53.8% of girls between 11- and 18-years-old report a sleep onset latency greater than 30min on school nights [4]. The prevalence of sleep deficiency and reduced sleep quality increases with age [4]. This poor quality and quantity of sleep in adolescents is concerning, given that insufficient sleep, reduced sleep quality, and irregular sleep patterns have been associated with various short and long-term physical and mental health consequences [5]. Therefore, intervention programs targeting unhealthy sleep in early adolescence (13–16 years old) are called for.

Only few available primary prevention interventions promoting healthy sleep in adolescents were successful in increasing sleep time [6] in the short term. However, these interventions were not able to maintain this effect in the long term [7] (see [8, 9] for exceptions) [8, 9], nor did they have any effect on sleep quality [6]. An important prerequisite to developing an effective healthy sleep intervention is to identify the most important and changeable factors that are related to adolescents’ sleep. Extensive survey research has already been conducted regarding both behavioral and environmental factors related to adolescents’ sleep and reported that screen time, physical inactivity, caffeine intake, tobacco, alcohol use, noise, traffic, pollution and neighborhood disorder are inversely associated with sleep duration [10–12]. However, very little research has been conducted into possible psychosocial factors (i.e., knowledge, attitude, perceived norms, perceived behavioral control, barriers and facilitators) related to adolescents’ sleep. The few studies that examined psychosocial factors only focused on one factor (i.e. perceived norms [13]), whereas behavioral change theories show that it is important to focus on multiple factors of health behavior to understand and change behavior [14, 15]. In addition, these studies had limited sample sizes [16, 17]. In a Canadian pilot study using standardized scripted interviews (N = 18), 15-year-old adolescents with a middle to high socio-economic status showed no insight regarding the long-term consequences of sleep deficiency and reported emotions to be the most important barrier of healthy sleep. Furthermore, parents and peers were identified as important influencers of their sleep [17]. A focus group study conducted in the UK (N = 33) showed similar results: adolescents from the 2nd year of high school (aged 13–14) acknowledged the influence that peers and parents have on their sleep and identified the use of electronic devices and the resulting dependency on them, particularly at night, as barriers for healthy sleep [16].

Interventions promoting healthy sleep in adolescents should, in addition to targeting the most important factors, actively involve adolescents in the development of the aforementioned intervention. Previous research has shown that involving the target group in intervention development and implementation ensures that intervention strategies are tailored to their needs and perceived as relevant, which increases the chance of effectiveness and sustainability [18]. Participation might be especially important in adolescents as they have a strong feeling of self-determination and autonomy [19]. However, no previous studies have investigated whether adolescents would be willing to change their sleep and whether or not they would be interested in being involved in the development and implementation of a healthy sleep intervention.

The purpose of this research was to perform focus group interviews with 13- to 16-year-old Flemish adolescents to collect in-depth information on the psychosocial factors related to their sleep, to investigate their willingness to participate in the development and implementation of a healthy sleep intervention and to explore their initial ideas regarding an intervention.

## Methods

### Protocol

A large school in East-Flanders (Flanders, Belgium) offering vocational, technical as well as general secondary educational tracks was recruited via convenience sampling. The principal of the school was contacted and gave permission to perform the study at the school. To assure maximum diversity in the sample, the principal was asked to randomly select one class from every grade (8th, 9th and 10th grade, respectively 13- to 14-year-olds, 14- to 15-year-olds and 15- to 16-year-olds) and from each educational track (vocational, technical and general). When class groups were too small (less than fifteen pupils), two classes were selected from this grade and educational track. Parents of pupils from the selected classes received a passive informed consent form one week before the commencement of data collection. Adolescents who had obtained parental consent, were verbally informed regarding the details of the study by the researcher and were asked to actively assent to participate by signing an informed assent form. Pupils were instructed to complete an online screening questionnaire regarding their demographics and their sleep duration and quality under the supervision of the researcher (AV, female, MSc in Health Education and Health Promotion; Doctoral Researcher). Two weeks later, focus group interviews were performed at school and during the regular school hours. For organizational purposes, the principal requested that each focus group interview consisted of students from the same class group, and for the number of focus group interviews to be decided upon before the start of the study. Based on previous experiences, the researchers (AV, and supervisors BD and MV (both female, PhD in Physical Education)) made the assumption that five or six focus group interviews would be sufficient to reach data saturation. However, to ensure maximum diversity in the sample, the researchers decided to organize nine focus group interviews, to ensure that students could be selected from each grade (8th, 9th and 10th ) and for each educational track (vocational, technical and general). Pupils were selected by the researcher (AV) based on the answers they gave in the screening questionnaire to guarantee maximum variability in sleep duration and quality and sex (i.e. girls/boys reaching/not reaching the sleep norm of 8h per night and girls/boys with sleep quality above/below the median). The researcher also selected a small number of additional pupils in case of absence or refusal to participate. The aim of the focus group study was explained to all participants prior to the interviews. AV moderated the interviews while LB or JJ (both female, MSc in Health Education and Health Promotion) assisted by observing, making notes and ensuring that the moderator did not overlook any participants who wanted to comment. Focus group interviews lasted 30 to 45min on average and followed a predetermined interview guide (see below). All interviews were audio-recorded after consent was obtained from the adolescents. Data collection took place between January and February 2017. All methods and procedures of this study were in accordance with the Declaration of Helsinki and were approved by the medical ethical committee of Ghent University (January 4, 2017; B670201630656).

### Measures

An initial screening questionnaire (see supplemental materials) was used to select adolescents for participation in focus group interviews with a variety of sleep duration and quality and sex (i.e. girls/boys reaching/not reaching the sleep norm of 8h per night and girls/boys with sleep quality above/below the median). The questionnaire was based on existing validated questionnaires and assessed sleep duration [20], sleep quality [21], daytime sleepiness [22], age, sex and educational track. Sleep duration was calculated by subtracting the sleep onset time from the wake-up time. A total score out of 60 was calculated for sleep quality and a score out of 32 for daytime sleepiness.

### Interview guide

The interview guide was developed based on a theoretical model of behavior change: the Reasoned Action Approach Model (RAAM) [23]. This model states that attitudes, perceived norms and perceived behavioral control towards a behavior, determine the intention to perform the behavior. The actual behavioral control that determines whether an intention is translated into actual behavior, is determined by knowledge, skills and environmental affordances and constraints [23]. The factors defined by the RAAM were used to draft the interview guide. The guide started with two opening questions on sleep duration and quality, and the knowledge of sleep norms and sleep hygiene, which allowed the participants to familiarize themselves with the topic of the focus group discussions. Transition and key questions were used to direct the discussion towards associated factors of sleep (e.g., knowledge and attitudes, perceived norms, perceived behavioral control, barriers). Following this first set of key questions, a second group of key questions mapped the opinion of adolescents towards being involved in developing and implementing a sleep intervention. The interview guide was a priori tested in a group of eight adolescents (13–16 years old). Seeing as adolescents understood all questions (e.g., no questions needed reframing, answers were to the point) and as the interview was not perceived to be too lengthy (35min), the interview guide was not adjusted. The aim of this pilot test was to check the adolescents’ ability to understand the questions and whether or not they perceived them as acceptable, therefore, the answers given in the test interview were not included in the final data set. The interview guide remained unchanged for the duration of all focus groups. An overview of the interview guide can be found in Table1. During the focus group discussions, the moderator followed the interview guide but used probes to obtain more in-depth information and demonstrated flexibility to allow for open discussions between pupils.

**Table 1:** Interview guide

**Opening question** 1. From the questionnaire we saw that you sleep 8h a night on average. Do you think this is enough? How many hours do you think you should sleep to get enough sleep? 2. What can you do to sleep well? What is good sleep hygiene?
**Transition** 3. How much do you think your peers sleep? How well do you think your peers sleep? 4. Why is it important to get enough and qualitative sleep?
**Key questions part 1** 5. What factors influence your sleep duration? = what actually makes you sleep enough or too little? 6. Would you like to change your sleep duration? And your sleep quality? How important would that be for you? Do you think you would be able to change it? Why or why not? 7. What do you think will change when you sleep more / better? Only advantages or also disadvantages? 8. What do you think you can do to change this? 9. What obstacles would there be to change this? What would make it difficult for you to change this? Think of personal obstacles, but also impeding factors in the environment (in your bedroom, house, street, influence of your family, …) 10. What could help you to tackle those obstacles (difficulties)?
**Key questions part 2** 11. Suppose we want to create some kind of intervention / health program / campaign that encourages you to sleep better and more, would you like to help develop this program? (if necessary, indicate the concept of an intervention using an example of another intervention related to sport) 12. How would you like to make such a program completely by yourself / independent, together with a number of peers? Would you like that, would you find it interesting, useful, important? 13. If you are fully responsible for developing the program, this would not only mean inventing the program, but also carrying it out, evaluating it afterwards, … Is that something you could do? Or would you need help from certain people? 14. Do you think it necessary that you have such a big / important role? Why or why not? 15. How would you like to do this at school? E.g. Create such a program with a number of students from your class / year / school and then implement it at school? If not at school, where else? 16. Do you already have some ideas for a campaign?
**Ending questions** 17. From what has been said, what is most important to you? What do you think I should definitely remember from this conversation? 18. Is there anything else you want to say?

### Analysis

Descriptive analyses on the questionnaire data were performed using IBM SPSS Statistics 23. NVivo 11 was used for structuring the data from the focus group interviews and thematic analysis [24] was used for data analysis. Two researchers (LB and JJ) independently coded the interviews, during and after data collection. Coding was partially inductive and deductive, in line with the hybrid approach of inductive and deductive thematic analysis as described by Fereday & Muir-Cochrane (2006)[25]. The researchers (LB and JJ) assigned open inductive codes to fragments but also deductively used the factors mentioned in the RAAM and other behavioral theories (i.e., barriers from the ASE-model [26]) as an inspiration for possible codes. Next, themes and subthemes were derived from the generated codes. A definition of identified psychosocial factors (discussed in the results as themes) can be found in Table2. The coders compared and debated their code nodes and trees. In the event of coding discrepancies, consensus was sought by involving a third researcher (AV). A final round of coding was performed by LB and JJ. LB, JJ and AV were trained in conducting data analysis in NVivo in the Master of Science in Health Promotion.

**Table 2:** Definition of factors from behavioral theories

Factor	Definition
Knowledge	The understanding one has of a key concept.
Facilitator	Factors that could facilitate the performance of the behavior.
Barrier	Factors that could limit the performance of the behavior.
Perceived behavioral control	Subjective probability that a person can execute a certain course of action.
Perceived norms	Beliefs about whether key people (e.g., family or friends) approve or disapprove of the behavior (normative beliefs).
Definitions based on Eldredge et al. (2016). Planning Health Promotion Programs: An Intervention Mapping Approach (15).	

## Results

### Descriptive characteristics

Eleven class groups with a total of 155 pupils were selected to fill in the screening questionnaire. Twelve pupils were absent during data collection. All pupils who were present (N = 143) had parental consent to participate in the study and actively assented to completing the online questionnaire. Nine focus group interviews (each including 8 pupils from a specific grade and a specific educational track) were performed during school hours. Descriptive characteristics from the focus group sample (N = 72) can be found in Table3. The average sleep duration reported by participants in the focus groups was 7h and 50min on weekdays and 9h and 45min on weekend days. Participants in the focus groups scored an average of 39.5 ( out of 60; higher scores reflect more positive sleep quality) on the short Adolescent Sleep Wake Scale (sleep quality) and 13.5 (out of 32; higher scores reflect higher levels of sleepiness) on the Pediatric Daytime Sleepiness Scale (daytime sleepiness).

**Table 3:** Descriptive characteristics focus group sample

**Mean age (± SD)**	14.8 (± 1.0) years	
**Sex** (%)	26 (36.1%) boys	
**Education** (%)	24 (33.3%) vocational 24 (33.3%) technical 24 (33.3%) general	
Mean score sASWS* (0–60) (± SD)	39.5 (± 7.6)	
Mean score PDSS** (0–32) (± SD)	13.5 (± 4.6)	
	**Week days**	**Weekend days**
**Mean sleep duration** (± SD)	7h 50m (± 1h 10m)	9h 45m (± 1h 17m)
**Mean time trying to fall asleep** (± SD)	22h 27m (± 1h 2m)	23h 52m (± 1h 28m)
**Mean sleep latency** (± SD)	28m (± 24m)	24m (± 30m)
**Mean wake time** (± SD)	6h 49m (± 25m)	10h 13m (± 27m)

*Short Adolescent Sleep Wake Scale: The higher the score, the better the sleep quality **Pediatric Daytime Sleepiness Scale: The higher the score, the more sleepiness experienced

### Factors related to sleep

Below, the most important themes and subthemes from the focus group interviews are presented. Themes are presented as a title; subthemes are indicated with a bold font. The major themes involving identified factors of sleep are defined in Table2 in the methods section. Due to practical considerations the number of focus group interviews was decided upon before the start of the study. No new information was obtained after analyzing the 5th focus group interview, meaning data saturation was reached. Nevertheless, all nine interviews were analyzed.

#### Knowledge about recommended amount of sleep, sleep hygiene and health benefits of sleep

Adolescents had different opinions on what the **recommended amount of sleep** is, ranging from seven to twelve hours.

“I think we should sleep eight or nine hours.” (9th grade technical education, boy).

“Seven to eight hours.” (9th grade technical education, boy).

“Eleven.” (9th grade vocational education, girl).

Most adolescents had a correct representation of what good **sleep hygiene** consists of. Nonetheless, several adolescents still experienced some misperceptions regarding good sleep hygiene, such as considering the performance of sports right before bedtime as a good practice. Moreover, additional aspects of sleep hygiene such as adjusted room temperature were not mentioned.

“Don’t watch TV half an hour before you go to sleep.” (9th grade technical education, boy).

“Don’t use your cell phone while in bed, or something like that.” (8th grade general education, boy).

“Do not drink Coca-Cola or eat and drink something with a lot of sugar.” (10th grade general education, boy).

The most important misperception on sleep hygiene was the idea of ‘catching up’ sleep during the weekend by sleeping in. A lot of adolescents indicated that they slept in during weekends, as a response to the fact that they do not have to wake up for school.

“I always try to catch up on sleep during the weekend and then I always think it’s alright again.” (10th grade vocational education, girl).

“During weekends you are allowed to sleep as long as you want, during weekdays you have to get up in the morning for school.” (9th grade vocational education, girl).

Finally, adolescents mostly talked about the **short-term benefits** as possible advantages of a sufficient amount of sleep (such as being energized, concentrated, better moods and memorizing), and not or to a lesser extent about **long-term effects** of poor sleep.

“You can concentrate well, you don’t get sick so quickly.” (10th grade technical education, girl).

“It is important to sleep well, to feel good about yourself.” (9the grade technical education, girl).

“You are fit to pay attention the next day.” (9th grade general education, girl).

“When you do not sleep enough, you are moody, which is annoying for other people.” (10th grade general education, girl).

#### Facilitators

Some adolescents indicated their smartphone as a facilitator of falling sleep. Others listened to music (on their smartphone) or read a book to fall asleep more easily.

“That is why people are on their smartphone for a longer time, that’s true for me anyway, I’m using my phone to get to sleep and then it’s pretty late before I sleep.“ (9th grade general education, boy).

When asked what they felt would help them to sleep better, several suggestions were given, such as leaving the smartphone downstairs, being physically active during the day, reading a book or setting an alarm which signals bedtime.

“I think if I would leave my cell phone downstairs I would get to sleep better and faster.” (10th grade general education, boy).

#### Barriers

Several barriers of healthy sleep were mentioned by adolescents, ranging from behavioral factors (such as screen time) to environmental factors (such as the starting time of schools) or emotional factors (such as ruminating).

All participants agreed that **smartphones** are the most important barrier to reaching a sufficient amount of sleep. Adolescents indicated that especially chatting (individual or in group conversations) stops them from going to sleep because they do not want to miss the further course of the conversation. They also mentioned losing track of time whilst texting or playing games on their smartphone. Finally, adolescents indicated that they would prioritize chatting over sleeping.

“The mobile phone is the main reason why I sleep late. I am on Facebook and all.” (10th grade technical education, boy).

“Yes, in the evening there are just so many people who text me and more is happening.” (10th grade technical education, girl).

“It would be better if I slept after 10 o’clock but I never succeed, so then I play something on my mobile, but then it quickly turns 11 o’clock or 12 o’clock.” (10th grade general education, boy).

“If you have to go to sleep earlier than your friends for example, then you are already sleeping while everyone is still sending messages or talking.” (8th grade technical education, girl).

Like conversations on smartphones, the fear of missing programs on **television** also influences adolescent’s bedtime. Adolescents said they find it annoying to miss TV programs, as they cannot join their peers who discuss the program at school the next day.

“Yes, and if you record a program, there are many friends who have already watched it, you cannot join the conversation and then it is no longer useful to watch it afterwards.” (10th vocational education, boy).

Also new technologies such as **Netflix** were mentioned as alternatives to television and as barriers.

“Yes, I watch Netflix, so I often lose track of time.” (10th vocational education, boy).

Adolescents also reported **leisure activities** as barriers to reach a sufficient amount of sleep, and reported that they were prioritized over healthy sleep.

“If you have to be somewhere until a quarter past eight or nine o’clock and then you still have to go home and wash yourself, it will take a long time until you are finished.” (9th grade general education, girl).

“Like your weekly sport activity or something, you hang out a bit longer or drink something in the canteen.” (9th grade general education, girl).

“Yes, if I had to go to sleep at nine, I would have to stop gymnastics and I don’t want to.” (9th grade technical education, boy).

Not all adolescents but a vast majority of young people indicated that **schoolwork** had an impact on their sleep. Due to the high amount of schoolwork, adolescents indicated that they go to bed later and that they experience more stress, resulting in increased difficulty falling asleep.

“Schoolwork, that’s why I go to bed later.” (10th grade general education, boy).

“Yes, especially stress actually. The pressure to get really good points at school that completely determines your life, you’re thinking about it a lot.” (10th grade general education, boy).

“I go to sleep too late because we have too much homework.” (8th grade general education, girl).

In line with this, **worrying or ruminating** was also mentioned by some participants as a barrier to falling asleep at an appropriate time.

“Yes, sometimes worrying.” (…) “What happened during the day or something like that” (10th grade vocational education, girl).

In addition, the **starting time of school** was also experienced, by some adolescents, as a barrier to reaching a sufficient amount of sleep.

“I sleep too little because I have to get up for school.” (10th grade general education, boy).

“I know, just let school hours start a little later… Then we would have more time to sleep, we would wake up faster and now we must be here at eight o’clock… That’s way too early” (8th grade general education, boy).

Finally, **noise** created by siblings in the room, parents, neighbors or environmental noise was mentioned as a barrier for high quality sleep.

“If they are playing music that is super loud, I have trouble sleeping.” (9th grade technical education, girl).

“Gosh yes, I always hear sounds. For example, in my room, sometimes the radiator ticks.” (8th grade technical education, girl).

#### Perceived behavioral control to change sleep

Most adolescents indicated that it would be hard to change their sleep. They felt that they would not be able to sleep if they would go to bed earlier, assuming that the sleep latency time would extend. Some adolescents also indicated that they would feel embarrassed telling peers that they want to sleep instead of chatting.

“If you tell people to go to bed an hour earlier, it would be almost impossible in the first few weeks because they are used to going to sleep much later… You would definitely lay awake.” (9th grade technical education, boy).

“If you suddenly get into bed at nine o’clock you can’t sleep either.” (10th grade general, boy) “No, because you are so used to going to sleep at ten and getting up at seven and if that suddenly changes, that will not work.” (10th grade general education, boy).

“I really have no discipline to go to bed earlier” (8th grade general education, boy).

“If you’re having a conversation and then have to say, I’ve got to sleep and it’s nine o’clock or something, that’s a little embarrassing to me.” (8th grade technical education, girl).

#### Perceived norm

Although adolescents found it difficult to estimate each other’s sleep, they assumed that their peers did not sleep enough and rated the sleep of their peers as poor. In addition, they assumed that their peers had a long sleep latency time.

“I think the others sleep eight or nine hours.” (8the grade general education, boy).

“Less for sure, seven hours or so.” (9th grade vocational education, girl).

“Eight hours is too much, if you ask around in our class. Most of them sleep six or seven hours or so.” (10th grade technical education, girl).

“I think we all sleep too little.” (8th grade general education, girl).

“I think it’s hard for peers to fall asleep.” (9th grade general education, girl).

According to the participants smartphones were the main reason for the poor perceived sleep of their peers.

“Researcher: and how well do you think they sleep?” “All: not good” “Researcher: Why do you think so?” “Because of smartphones and electrical devices.” (10th grade vocational education, girl).

#### Family support: family rules

Several adolescents mentioned family rules regarding bedtime as a factor influencing their sleep. For some adolescents, a fixed bedtime was also accompanied by handing over the smartphone to the parent(s) when going to bed. Although some adolescents acknowledged these rules as having a positive influence on their sleep, others indicated that these sometimes provoked feelings of irritation or frustration which then had a negative effect on their ability to fall asleep. Adolescents thought it would be easier if the rules were mutually discussed in advance.

“My parents tell me to leave my smartphone downstairs.” “Researcher: And does that help?” “Yes, I think so because otherwise I would continue to send text messages and now I have to go to sleep at some point, otherwise I would keep texting and fall asleep much later.” (10th grade general education, girl).

“My mom used to take my cell phone and I became so annoyed about it that I couldn’t sleep either. Suppose you want to send something, and she takes it away, then it’s just like you are ignoring someone, which is an annoying feeling because you were not able to finish the conversation.” (9th grade technical education, boy).

#### Involvement in the development (and implementation) of a healthy sleep intervention

Adolescents had a positive **attitude towards being involved** in the development and implementation of a healthy sleep intervention. This was considered interesting and important by the adolescents, and they mentioned that it would help them improve their own sleep. Furthermore, they indicated that it would be essential for them to share their opinion, since they are most able to advocate what is interesting and important for adolescents.

“When it comes to youth, it is important that we say what we think.” (10th grade general education, boy).

“I would help, because it will make yourself better and others will also benefit.” (9th grade general education, girl).

However, they had a low sense of **self-efficacy** to complete this task autonomously. Consequently, they felt that it wasn’t necessary for them to play the most important role in the entire process. In addition, adolescents felt unable to do this independently due to a lack of experience and expertise, and being too young to take on such responsibility. They indicated that it was important that an older person with more experience and knowledge (such as people connected to the university, teachers, school management or their parents) would guide them through the process.

“I would not know what needs to be done.” (…) “If it is a lot of work, then I don’t want to do it, because I am someone who wants to do everything well.” (9th grade general education, girl).

“Yes… we need guidance.” (10th grade general education, girl).

#### Sleep intervention ideas

When asked if they could already generate some **ideas** for this hypothetical **intervention**, participants came up with several ideas: a quiz on sleep, a competition between class groups to sleep the most, rewards when performing some tasks, setting a goal, developing an application to monitor sleep or sleeping as much as possible with the intent of raising money for a charity.

“An app or something, then you can always fill it in and receive feedback.” (10th grade general education, girl).

The students unanimously agreed that school would be the ideal **setting** for a sleep intervention, because of the already existing bond between the students and the fact that young people are easily accessible at school.

“I also think it would be good to do it at school, you know everyone, you see each other every day, I think that’s better than with people you don’t know.” (9th grade general, girl) “Yes, you can help each other. (9th grade general education, girl)

“Yes, I think so, because it is an assembly point of young people that could use some advice on healthy sleep.” (10th grade general education, boy).

## Discussion

The goal of this study was to explore perceived psychosocial factors related to sleep in 13- to 16-year-old Flemish adolescents and to investigate their willingness to participate in the development and implementation of a school-based healthy sleep intervention.

Adolescents in this study confirmed that smartphones are the main reason for a delayed sleep time. Next to time displacement as indicated by the adolescents, literature also suggests psychological stimulation and the effect of blue light emitted from screens as underlying mechanisms of the influence of screen time on sleep [27]. Some adolescents mentioned using their smartphone as a medium to fall asleep, suggesting that the influence of smartphone use on sleep can be both positive and negative [28]. Although adolescents considered a good night sleep to be important, they did not prioritize it over smartphone use. Research has suggested that adolescents value the short-term benefits of screen time more than healthy sleep [29]. Indeed, showing a stronger preference for short-term rewards over long-term rewards is typical for adolescents [30]. As adolescents also prioritized other activities such as watching television or leisure activities over sleep, future interventions could focus on the prioritization of sleep. Attitudes towards sleep could be enhanced by highlighting the advantages of more and better sleep and less screen time for example. This study confirmed that adolescents have no insight into long-term consequences of poor sleep [17]. It is important that adolescents recognize the increased individual short- and long-term benefits associated with healthy sleep as compared to screen time in bed or close to bedtime. However, past research shows that an increase in knowledge alone is not sufficient to enhance these attitudes and change sleep [31, 32]. Specific evidence-based behavior change techniques targeting attitudes are needed. Examples of such techniques are ‘direct experience’ (e.g., encouraging students to avoid screen time at bedtime and evaluate their sleep at the end of the week) or ‘arguments’ combined with ‘cultural similarity’ (e.g., showing a video of a peer on Instagram talking about the benefits of avoiding screen time at bedtime)[15].

The present study suggests that several actions could be taken by schools to improve sleep in adolescents. First of all, the time at which schools start could be delayed, seeing as this was mentioned by adolescents as an important barrier for having a sufficient amount of sleep. From a physiological perspective, adolescents often experience a delayed sleep phase due to hormonal fluctuations and a changed circadian rhythm which is associated with a significant decrease in melatonin production [33]. However, societal demands such as early school start times [34] remain unchanged, this consequently leads to a reduced amount of sleep and sleep deprivation on weekdays. Participants mentioned they compensated this reduced sleep time on weekend days by sleeping in. Nonetheless, sleeping late on weekends disrupts the sleeping pattern, which in turn can increase sleep onset latency time on Sunday evening and daytime fatigue and sleepiness on the Monday and Tuesday of the following week [35]. Delaying school start time would be beneficial for the quantity of sleep of many students, and consequently their overall well-being [36]. International studies with delayed school start times demonstrated a significant increase in the amount of sleep, even with minimal delays of half an hour [37]. Further, schools could be made aware of the impact that large amounts of schoolwork have on their pupils; causing stress and worrying which can delay sleep latency time. Earlier research showed that experiencing a high amount of school pressure is associated with a decrease of fifteen minutes in total sleep duration on school days and an increase in sleep onset difficulties [38]. However, next to ruminating about schoolwork, adolescents also worried about other things (e.g., friends) or were kept awake by disturbing noises. As adolescents perceived a long sleep latency time as unpleasant, future interventions could provide tools that help adolescents to overcome this delay, such as an app coaching and supporting them in maintaining healthy sleep hygiene (i.e. regular bed and wake times, limiting evening screen time, limiting caffeine and sugar intake after 16h, maintaining a comfortable sleep environment) or meditation or mindfulness group lessons [39].

Future sleep interventions should take the reported low behavioral control towards improving sleep and decreasing screen time into account. Adolescents believed that they would be awake for a long time when going to bed at an earlier time. Recent research shows that it takes two weeks to change existing sleep patterns when strictly following sleep hygiene instructions [40], as such, suggesting persistence and slowly building towards a healthier sleep time should be a key message. Several reviews identified four weeks as a common duration for school-based sleep education programs [7, 31],however, a range of psychosocial factors (i.e., attitudes, perceived norms, perceived behavioral control) need to be targeted to achieve lasting behavior change alongside knowledge. Consequently, a longer intervention duration than four weeks would be needed. Other successful health-promoting interventions at school focusing on diet and physical activity for example, lasted an entire school year [41].

Adolescents’ sleep is influenced by both friends and parents. Even though several adolescents indicated finding sleep important and mentioned leaving their smartphone downstairs when going to bed, adolescents still perceived a norm of unhealthy sleep and excessive smartphone use in bed. As research shows that positive peer influence can protect adolescents from risky health behaviors [42], future interventions could normalize the perceived norm and create a positive culture regarding sleep. This could be achieved by providing opportunities for social comparison and by modelling, more specifically, bringing the message of healthy sleep through influencers to adolescents [15]. In contradiction with the findings of Gruber and colleagues [17], adolescents indicated that they would find it hard to tell peers they wanted to sleep instead of chat. Sleep interventions might thus encourage adolescents to resist the peer pressure to go to sleep late, for example by using the behavior change technique ‘public commitment’ (e.g., a contract is signed by the whole class group declaring they will not text each other after 9 pm) [15]. Future interventions could also involve parents and encourage them to set rules concerning sleep, in mutual agreement with the adolescents to reduce feelings of frustration.

Finally, it could be important to actively involve adolescents in the development of a healthy sleep intervention. Adolescents have a better understanding, than adult researchers, of their circumstances and how to best influence their peers. The focus groups revealed that adolescents would like to be involved in the development of an intervention, but pointed out that it would be hard to do this without the assistance of an adult, be it a teacher, parent or researcher. Applying a participatory approach in which researchers and the target group actively collaborate throughout the research process could provide a solution. In this kind of research, the researchers and the target group are considered as equals, resulting in a more valuable outcome [43, 44]. Adolescents and researchers could co-create a school-based healthy sleep intervention by following the various steps of intervention planning together. If there is evidence for its effectiveness, this intervention could be scaled up following the cascade model as described by Leask et al. (2019). This model suggests that a locally developed intervention could be transported and adapted in collaboration with or by a new group of local stakeholders and end-users for the same purpose, in a different setting [43]. A short participatory process could be set up for this. Earlier research showed promising results when applying a participatory approach to promoting healthy sleep in school-aged children (7–11 years-old), with an increase of eighteen minutes in sleep duration [45]. According to adolescents, school would be the ideal setting for a healthy sleep intervention, as it assembles a heterogeneous group of adolescents. The school setting provides unique opportunities for health research with adolescents: the target group is easily reached and school-based interventions are considered cost-effective [29]. Furthermore, it provides the opportunity to include various important environmental actors (such as parents, peers and school staff) in the intervention.

Some study limitations need to be acknowledged. Focus group discussions provide the opportunity to elaborate on topics, but they might cause socially desirable answers. However, the moderator emphasized that all answers and comments were correct and valuable. Furthermore, this study cannot establish a causal relationship between the identified related factors and sleep, nor can the strength of the relationships be determined, suggesting that quantitative longitudinal or experimental research is required. However, this study offers a basis for such research, as the results could be used to formulate hypotheses in future research. Additionally, although the study included a variation of different participants (different types of education, age, sex, sleep patterns) only one school was included in this study and no information on ethnicity was collected. This might limit the generalizability of the study findings and the suggestions for future interventions. Finally, the factors mentioned in this research were socio-cognitive, while unconscious factors (i.e. impulsive, such as habit or mood) may also play a role in healthy sleep [46].

## Conclusion

Future interventions promoting healthy sleep in adolescents could focus on enhancing knowledge concerning sleep guidelines, sleep hygiene (especially maintaining a regular sleep pattern) and the long-term consequences of sleep deficiency, prioritizing positive attitudes towards sleep over positive attitudes towards screen time, finding solutions for barriers towards healthy sleep such as ruminating or early school start times, increasing perceived behavioral control and creating a positive perceived norm regarding healthy sleep. The involvement of environmental actors such as peers, parents and school staff would facilitate targeting these factors, as such the school setting would be ideal for a healthy sleep intervention. Finally, involving adolescents in intervention development would be beneficial, seeing as they indicated that they are the most adequately informed individuals concerning their own circumstances.

## Electronic supplementary material

Below is the link to the electronic supplementary material.

Supplementary Material 1

Supplementary Material 2

## Data Availability

The data used and/or analyzed during the current study are available from the corresponding author on reasonable request.
