# Supplementary material for: Psychosocial factors related to sleep in adolescents and their willingness to participate in the development of a healthy sleep intervention: a focus group study
Source: BMC Public Health. 2022 Oct 7;22:1876. doi: 10.1186/s12889-022-14278-3 (PMC9547416; doi:10.1186/s12889-022-14278-3)
Supplement: Supplementary file 1 — Supplementary Material 1 [file 12889_2022_14278_MOESM1_ESM.docx]

**S1 Questionnaire**

Dear student,

This questionnaire assesses your sleeping behavior from the past month. Please indicate the answer that suits you best. There are no right or wrong answers, try to be as honestly as possible. It is also not necessary to dwell on the question for a long time, often the answer that comes to mind first is best.

Thanks in advance!!

1. My first name is ………………………………………. My familyname is ……………………………………………..

2. I am a ... O Boy

O girl

3. I was born on ………… (day) in the month …………………… .. of the year …………………………… ..

4. I am in… O Second grade

O Third grade

O Fourth grade

5. What type of education do you follow?

O vocational secondary education

O technical or art secondary education

O general secondary education

Here are some questions about 'going to sleep'. The 'process' of going to sleep is depicted in the image below. People often go to bed at a certain time (picture 1), but first stay awake in bed for a while (reading a book, checking smartphone, ...) (picture 2). Only after some time do they really get ready to sleep (picture 3). After a certain time they actually fall asleep (picture 4).

** The original questionnaire contained a series of pictures to illustrate the process of going to bed and fall asleep in 4 steps. These pictures have been removed to avoid any copyright infringement.*

6. The time when I go to bed (picture 1) varies from day to day.

**On school days On days off**

O I agree O I agree

O I disagree O I disagree

7. When do you usually get into your bed? (picture 1)

**On school days On days off**

O Before 20h O Before 20h

O Between 20h01and20h15 O Between 20h01 and 20h15

O Between 20h16 and 20h30 O Between 20h16 and 20h30

O Between 20h31 and 20h45 O Between 20h31 and 20h45

O Between 20h45 and 21h O Between 20h45 and 21h

O Between 21h01 and 21h15 O Between 21h01 and 21h15

O Between 21h16 and 21h30 O Between 21h16 and 21h30

O Between 21h31 and 21h45 O Between 21h31 and 21h45

O Between 21h46 and 22h O Between 21h46 and 22h

O Between 22h01 and 22h15 O Between 22h01 and 22h15

O Between 22h16 and 22h30 O Between 22h16 and 22h30

O Between 22h31 and 22h45 O Between 22h31 and 22h45

O Between 22h46 and 23h O Between 22h46 and 23h

O Between 23h01 and 23h15 O Between 23h01 and 23h15

O Between 23h16 and 23h30 O Between 23h16 and 23h30

O Between 23h31 and 23h45 O Between 23h31 and 23h45

O Between 23h46 and 00h O Between 23h46 and 00h

O Between 00h01 and 00h15 O Between 00h01 and 00h15

O Between 00h16 and 00h30 O Between 00h16 and 00h30

O Between 00h31 and 00h45 O Between 00h31 and 00h45

O Between 00h46 and 1h O Between 00h46 and 1h

O Between 1h01 and 1h15 O Between 1h01 and 1h15

O Between 1h16 and 1h30 O Between 1h16 and 1h30

O Between 1h31 and 1h45 O Between 1h31 and 1h45

O Between 1h46 and 2h O Between 1h46 and 2h

O Between 2h01 and 2h15 O Between 2h01 and 2h15

O Between 2h16 and 2h30 O Between 2h16 and 2h30

O Between 2h31 and 2h45 O Between 2h31 and 2h45

O Between 2h46 and 3h O Between 2h46 and 3h

O Between 3h01 and 3h15 O Between 3h01 and 3h15

O Between 3h16 and 3h30 O Between 3h16 and 3h30

O Between 3h31 and 3h45 O Between 3h31 and 3h45
O Between 3h46 and 4h O Between 3h46 and 4h
O 4h01 or later O 4h01 or later

**!! Attention: You filled in the hour when you crawl into bed, so you may stay awake for some time !! (picture 2)**

**8. When do you really get ready to fall asleep? (picture 3)**

**On school days On days off**

O Before 20h O Before 20h

O Between 20h01and20h15 O Between 20h01 and 20h15

O Between 20h16 and 20h30 O Between 20h16 and 20h30

O Between 20h31 and 20h45 O Between 20h31 and 20h45

O Between 20h45 and 21h O Between 20h45 and 21h

O Between 21h01 and 21h15 O Between 21h01 and 21h15

O Between 21h16 and 21h30 O Between 21h16 and 21h30

O Between 21h31 and 21h45 O Between 21h31 and 21h45

O Between 21h46 and 22h O Between 21h46 and 22h

O Between 22h01 and 22h15 O Between 22h01 and 22h15

O Between 22h16 and 22h30 O Between 22h16 and 22h30

O Between 22h31 and 22h45 O Between 22h31 and 22h45

O Between 22h46 and 23h O Between 22h46 and 23h

O Between 23h01 and 23h15 O Between 23h01 and 23h15

O Between 23h16 and 23h30 O Between 23h16 and 23h30

O Between 23h31 and 23h45 O Between 23h31 and 23h45

O Between 23h46 and 00h O Between 23h46 and 00h

O Between 00h01 and 00h15 O Between 00h01 and 00h15

O Between 00h16 and 00h30 O Between 00h16 and 00h30

O Between 00h31 and 00h45 O Between 00h31 and 00h45

O Between 00h46 and 1h O Between 00h46 and 1h

O Between 1h01 and 1h15 O Between 1h01 and 1h15

O Between 1h16 and 1h30 O Between 1h16 and 1h30

O Between 1h31 and 1h45 O Between 1h31 and 1h45

O Between 1h46 and 2h O Between 1h46 and 2h

O Between 2h01 and 2h15 O Between 2h01 and 2h15

O Between 2h16 and 2h30 O Between 2h16 and 2h30

O Between 2h31 and 2h45 O Between 2h31 and 2h45

O Between 2h46 and 3h O Between 2h46 and 3h

O Between 3h01 and 3h15 O Between 3h01 and 3h15

O Between 3h16 and 3h30 O Between 3h16 and 3h30

O Between 3h31 and 3h45 O Between 3h31 and 3h45
O Between 3h46 and 4h O Between 3h46 and 4h
O 4h01 or later O 4h01 or later

**9. How long (in minutes) does it usually take to fall asleep? (picture 4)**

On school days On days off

…. minutes …. minutes

Here are some questions about waking up and getting up. The process is depicted again in an image. First of all, one wakes up (picture 5), often people lie down for a while before they actually get up (picture 6).

** The original questionnaire contained a series of pictures to illustrate the process of waking up in 3 steps. These pictures have been removed to avoid any copyright infringement.*

**10. When do you usually wake up / average? (picture 5)?**

On school days On days off

O Before 5h00 O Before 5h00

O Between 5h01 and 5h15 O Between 5h10 and 5:15

O Between 5h16 and 5h30 O Between 5h16 and 5h30

O Between 5h31 and 5h45 O Between 5h31 and 5h45

O Between 5h46 and 6h00 O Between 5h46 and 6h00

O Between 6h01 and 6h15 O Between 6h01 and 6h15

O Between 6h16 and 6h30 O Between 6h16 and 6h30

O Between 6h31 and 6h45 O Between 6h31 and 6h45

O Between 6h46 and 7h00 O Between 6h46 and 7h00

O Between 7h01 and 7h15 O Between 7h01 and 7h15

O Between 7h16 and 7h30 O Between 7h16 and 7h30

O Between 7h31 and 7h45 O Between 7h31 and 7h45

O Between 7h46 and 8h00 O Between 7h46 and 8h00

O Between 8h01 and 8h15 O Between 8h01 and 8h15

O Between 8h16 and 8h30 O Between 8h16 and 8h30

O Between 8h31 and 9h O Between 8h31 and 9h

O Between 9h01 and 9h15 O Between 9h01 and 9h15

O Between 9h16 and 9h30 O Between 9h16 and 9h30

O Between 9h46 and 10h O Between 9h46 and 10h

O Between 10h01 and 10h15 O Between 10h01 and 10h15

O Between 10h16 and 10h30 O Between 10h16 and 10h30

O Between 10h31 and 10h45 O Between 10h31 and 10h45

O Between 10h46 and 11h O Between 10h46 and 11h

O Between 11h01 and 11h15 O Between 11h01 and 11h15

O Between 11h16 and 11h30 O Between 11h16 and 11h30

O Between 11h31 and 11h45 O Between 11h31 and 11h45

O Between 11h46 and 12h O Between 11h46 and 12h

O Between 12h01 and 12h15 O Between 12h01 and 12h15

O Between 12h16 and 12h30 O Between 12h16 and 12h30

O Between 12h31 and 12h45 O Between 12h31 and 12h45

O Between 12h46 and 13h O Between 12h46 and 13h

O Between 1h01 and 13h15 O Between 13h01 and 13h15

O Between 13h16 and 13h30 O Between 13h16 and 13h30

O Between 13h31 and 13h45 O Between 13h31 and 13h45

O Between 13h46 pm and 14h O Between 13h46 pm and 14h

O Later than 14h01 O Later than 14h01

**11. How long (in minutes) does it usually take to get up after you wake up? (picture 6)**

On school days On days off

…. minutes …. Minutes

**12. The time I get up (picture 6) differs from day to day.**

On school days On days off

O I agree O Agree

O I disagree O I disagree

**13. Are you awakened on school days (eg by alarm clock or by your parents)?**

O Yes

O No (go to question 14)

I**f YES, how often do you wake up BEFORE your alarm goes off / your parents wake you up?**

O never

O almost never

O sometimes

O almost always

O always

**15. Think back to the past month, to what extent do the following statements apply to you?**

|  | Never | Almost never | Sometimes | Often | Almost always | Always |
| --- | --- | --- | --- | --- | --- | --- |
| Before bed I do something that could keep me awake (such as gaming, watching TV, exercising or using a smartphone) |  |  |  |  |  |  |
| When it's time to go to sleep, I want to stay up and do other things. |  |  |  |  |  |  |
| I am ready to go to sleep when it is bedtime. |  |  |  |  |  |  |
| I try to postpone the moment I go to sleep. |  |  |  |  |  |  |
| When it is time to sleep, I struggle to relax. |  |  |  |  |  |  |
| I take medication to get to sleep. |  |  |  |  |  |  |
| I need help getting to sleep (eg I have to listen to music, watch TV, or need someone else in bed with me). |  |  |  |  |  |  |
| When I wake up at night, I have trouble getting back to sleep. |  |  |  |  |  |  |
| When I wake up at night, I'm tossing. |  |  |  |  |  |  |
| When I wake up at night, I need help to get back to sleep (eg I have to watch TV, read, sleep with another person). |  |  |  |  |  |  |
| When I wake up in the morning, I feel ready to get up. |  |  |  |  |  |  |
| When I wake up in the morning I feel rested and alert. |  |  |  |  |  |  |

**17. Some people feel sleepy during the day. How many daytime sleep problems have you had in the past month? Answer the questions below as honestly as possible by ticking one answer.**

|  | Never | Rarely | Sometimes | Regularly | Always |
| --- | --- | --- | --- | --- | --- |
| How often do you fall asleep or get sleepy in class? |  |  |  |  |  |
| How often do you get sleepy while doing your homework? |  |  |  |  |  |
| Are you usually alert most of the day? |  |  |  |  |  |
| How often do you feel tired and moody during the day? |  |  |  |  |  |
| How often do you have trouble getting up in the morning? |  |  |  |  |  |
| How often do you fall back asleep after waking up in the morning? |  |  |  |  |  |
| How often should someone wake you up in the morning? |  |  |  |  |  |
| How often do you think you need more sleep? |  |  |  |  |  |
| How often do you take naps during the day? |  |  |  |  |  |

**18. How would you rate your sleep quality from the last month?**

O Very good

O Good

O Poor

O Bad

O Very bad

**19. Do you consider yourself**

O a good sleeper

O a mediocre sleeper

O a bad sleeper
