# Supplementary material for: Psychosocial factors related to sleep in adolescents and their willingness to participate in the development of a healthy sleep intervention: a focus group study
Source: BMC Public Health. 2022 Oct 7;22:1876. doi: 10.1186/s12889-022-14278-3 (PMC9547416; doi:10.1186/s12889-022-14278-3)
Supplement: Supplementary file 2 — Supplementary Material 2 [file 12889_2022_14278_MOESM2_ESM.docx]

**Supplementary files legend**

Supplementary file 1

Questionnaire
